# Benzo[a]pyrene and Benzo[k]fluoranthene in Some Processed Fish and Fish Products

**DOI:** 10.3390/ijerph120100940

**Published:** 2015-01-19

**Authors:** Olatunde S. Olatunji, Olalekan S. Fatoki, Beatrice O. Opeolu, Bhekumusa J. Ximba

**Affiliations:** Department of Chemistry, Faculty of Applied Sciences, Cape Peninsula University of Technology, Cape Town, 8000, South Africa; E-Mails: FatokiO@cput.ac.za (O.S.F.); opeplub@cput.ac.za (B.O.P.); Ximbab@cput.ac.za (B.J.X.)

**Keywords:** benzo[a]pyrene, benzo[k]fluoranthrene, processed fish (fried, grilled and boiled), sum of the two PAHs (∑_2_PAH), abundance

## Abstract

In this study, the concentration levels of the probable carcinogenic PAH fractions, benzo[a]pyrene (BaP) and benzo[k]fluoranthrene (BkF) in fillets of some processed fish species were investigated. Fish species comprising *Merluccius poli* (hake), *Tyrsites atun* (snoek), *Seriola lalandi* (yellow-tail) and *Brama brama* (angel fish) were bought in fish shops at Gordon’s Bay, Western Cape, South Africa. The fish were gutted, filleted and prepared for edibility by frying, grilling and boiling. Polycyclic aromatic hydrocarbons were extracted from each homogenized fish sample, cleaned-up using solid phase extraction (SPE), and analysed for the PAH fractions, BaP and BkF using a Gas Chromatograph coupled with a Flame Ionization Detector (GC-FID). The sum of the two PAHs (∑_2_PAH)* i.e.*, BaP and BkF ranged between 0.56 and 1.46 µg/kg, in all boiled, grilled and fried fish species. The fried fish extracts showed significantly higher (*p* < 0.05) abundance of ∑_2_PAH, than grilled and boiled fish. Dietary safety and PAHs toxicity was also discussed.

## 1. Introduction

The demand for fish and fish products is increasing in most countries, especially in view of their enumerated nutritional health advantages of low fat and low cholesterol content compared with meat. They constitute a major proportion of human diet, which supplies essential proteins (amino acids) and other essential minerals such as zinc, iron, selenium, phosphorus, niacin, riboflavin, choline and vitamins [1,2]. Thousands of metric tonnes of several hundreds of fish species including freshwater fish and sea/marine fishes from rivers, seas and oceans are harvested through fishing and trawling activities, and consumed every year [3]. Aquaculture breeding which aims at increasing fish production, and allows the systematic production of fish, on small and commercial scale is now a common source of fresh water fish supply. Advances in animal husbandry, biotechnology and genetic have also facilitated high yield and constant supply.

There is usually the need to preserve catch or harvest to avoid decomposition in the time gap prior to consumption. In large commercial harvesting, refrigeration and ice freezing is common practice. The trend in selling fish and fish products with special savoury and organo-leptic profile dictated by different cultures, or in pre-packaged cuts has increased significantly [4]. The methods used in the preservation and processing of fish therefore vary between different traditions depending on the availability of the fishes, and these techniques also changes over time [3]. An early method of preservation, especially in subsistent harvesting in small sedentary communities involves smoke curing. In contemporary times, the methods used include boiling, grilling, roasting, braaing, and char boiling, depending on the perceptions of taste widely demanded in the market.

During the preparation of fish and fish products for dietary consumption and or preservation purposes, contaminants such as polycyclic aromatic hydrocarbons, heterocyclic aromatic amine and other contaminants are either derived within fish matrices or added to the fish. PAHs have been reported in food processed by methods involving heat treatment, especially grilling, char boiling, and braaing, Karl and Leinemann [5] reported that PAHs penetrate into smoked products, where they are protected from light and oxygen, and after some time, the concentration stabilizes at a certain constant level. According to Alcicek [6], the levels of PAH in smoked fish products increases drastically during the process of smoking, and this slightly decreases with time after smoke treatment due to light decomposition and interaction. Thus the direct and or indirect contact of fish with the flames or other heat/thermal sources, and the pyrolysis of the fats in the fish muscles result in the generation of PAHs [7]. As a result, the safety of fish and fish products is hampered by their association with PAHs.

The presence of PAHs in food poses quite serious concerns, because food is an important and direct source of human exposure. The consumption of fish and fish products especially those processed under high temperature such as smoking [8], grilling, and frying, has been linked to increase risk of immune-suppression, gene-toxicity, carcinogenic toxicity (such as cancer of the colon and rectum), and mutagenic toxicity [7,9]. Studies evaluating dietary intake levels of PAHs, especially intake concentration levels associated with normal and critical thresholds in human diet, and the major routes of exposure have been reported [10,11,12,13,14,15,16,17,18].

Data concerning the characterization of the safe dietary levels of PAHs in home prepared foods or those bought or consumed outdoor in pubs, food shops and restaurants in many communities in Africa and especially the Cape Town South Africa environment are scarce. There is the need to generate data in order to characterize the safety levels of PAHs in commonly harvested fish species with respect to the methods of preparation for human consumption. In this study, the concentration levels of the probable carcinogenic PAH fractions, benzo[a]pyrene and benzo[k]fluoranthrene in fillets of several fish species; *Merluccius poli* (hake), *Tyrsites atun* (snoek), *Seriola lalandi* (yellow-tail) and *Brama brama* (angel fish) were investigated, in order to determine their human dietary exposure levels, and to evaluate the effect of different methods of preparation (boiling, grilling and frying) on the resident concentration levels in the prepared fish fillets.

## 2. Experimental Section

### 2.1. Sample Collection

Four fish species: *Merluccius poli* (hake) (N = 30), *Tyrsites atun* (snoek) (N = 30), *Seriola lalandi* (yellow-tail) (N = 30) and *Brama brama* (angel fish) (N = 30) were bought over the counter from fish shops in Gordon’s Bay, Western Cape, South Africa. Gordon's Bay is a rural population town, situated around the old harbour and Bikini Beach, along the coastal line of the Atlantic Ocean. The leading occupation of the local population is largely fishing. Due to the serenity of the coastal line, Gordon’s Bay attracts an inflow of tourists and other visitors. There are a number of attendant restaurants and luxury hotels, catering for the needs of the tourists and visitors. Among the delicacies served in the restaurants, are different sea foods and fish species of varying savours.

### 2.2. Sample Preparation

The fish samples were gutted to remove their respiratory and gastro intestinal tracks (GIT), and filleted. The fillets of the different fish samples were tenderized using vinegar and lemon juice, and then spiced with salt, garlic powder, pepper and onions. Cheese and soy sauce was melted on each of the fish, and left to stand for about 15–20 min to ensure fillet penetration. The fish fillets (10 each of the different species) were thereafter prepared for consumption by boiling, grilling and frying, respectively. Fillets grilling was done in an electric oven for about 15 min. The samples for frying were embedded in cracker crumbs and eggs, and tartar sauce and fried in vegetable oil at temperature of about 375 °C for about 15 min. The boiling of the fish fillets was done in an ample quantity of water, for about 15 min.

About 30 g each of the fish samples prepared by grilling, frying and boiling were separately homogenized, using a pestle and mortar. Each of the homogenised fish gravies was packed in foil papers and inserted into well labelled zip polypropylene bags, and stored in the refrigerator until analysis. Analysis was conducted within three days of each sample collection and preparation.

### 2.3. Extraction of PAHs (Liquid-Liquid Extraction)

Five g each of the homogenized fish fillets was separately weighed into different pre-cleaned beakers. Anhydrous Na_2_SO_4_ (2 g) was added to each, and then mixed to remove all traces of water, and hydrolyzed in methanolic KOH (100 mL, 9:1 v/v) [19,20]. This was placed on a horizontal shaker for about 20 min, and then refluxed in a water bath at 60 °C for 1½ h. Polycyclic aromatic hydrocarbon was thereafter extracted into a 1:4 mixture of dichloromethane (DCM) and *n*-hexane.

### 2.4. Sample Clean up

The PAH extracts were cleaned a using solid phase extraction (SPE) column, containing neutral/basic/acidic/neutral silica. Each of the SPE columns were activated by running 15 mL of DCM through the column at a rate of 1 mL/min, followed by 20 mL *n*-hexane. About 15 mL of a 1:4 mixture of DCM and *n*-hexane was thereafter run through the column and allowed to drop and level up on the solid phase column. The fillets PAH extracts were loaded on each of the column and eluted at a flow rate of 1 mL/min. Each of the SPE columns was washed with 20 mL *n*-hexane after the elution of the fillet extracts to recover the residues of PAHs. The eluent were combined, transferred into separate test tubes, centrifuged at 2000 rpm for 20 min and decanted into clean beakers. Each of the eluents were dried under nitrogen stream and reconstituted in *n*-hexane GC for analysis.

### 2.5. Analysis

The separation and determination of the PAH fractions in the PAH extracts in hexane was carried out as described earlier by Olatunji* et al.* [20]. Briefly, the fried, grilled and boiled fish fillet PAH extracts in *n*-hexane were separated on a ZB-5MS column (Agilent USA: 30 m, 0.25 mm i.d. × 0.25 µm film thickness) in a helium stream, using a gradient temperature programme, and quantified for the fractions BaP and BkF by Flame Ionization Detection (GC-FID-Shimadzu 2010 *plus*, Japan). The gradient temperature programme for separation was set at an initial temperature of 80 °C and held for 4 min. A ramp rate of 8 °C/min was used to raise the temperature to 170 °C, held for 10 min and then to 250 °C at the same rate, and held for another 10 min. A further increase to 300 °C was achieved at a ramp rate of 10 °C·min^−1^ and held for 10 min. The injector and detector temperature were set at 280 °C and 320 °C respectively. Response factor for BaP and BkF was measured and calculated at thrice before analysis using individual internal standard, and as checks in between and at the end of each batch of 10 sample GC injections.

### 2.6. Standard Preparation

Stock solution of benzo[a]pyrene and benzo[k]flouranthene (1000 mg/L) were prepared by dissolving neat crystals of benzo[a]pyrene, and benzo[k]fluorantene (0.01 g) in a 10 mL 1:4 mixture of DCM and *n*-hexane. The working solutions of the standards in the range 5–50 µg/L were then prepared from the stock solution, for instrument/method calibration.

## 3. Results and Discussion

### 3.1. Performance of Method

The limit of detection (LOD) was defined by the quantity, three multiples of the standard deviation (SD) of mean of blank signal (S/N signal ratio) of 10 blank measurements. The limit of quantification (LOQ) reflects the probability density function, 10 multiples of the mean of SD of mean of signal to noise ratio (S/N) for 10 normally distributed measurements at the blank, in comparison to the baseline noise. The LOD and LOQ for BaP and BkF were 0.1 µg/kg and 0.3 µg/kg, respectively.

The linearity of the instrument response to increasing working standard concentration was determined to range between 0.1–42.5 µg/kg for five set calibration standards (5–50 µg/kg) in a 1:4 mixture of DCM and *n*-hexane. The coefficient of regression (R^2^) obtained for the PAH fractions were; BaP, 0.9872 and BkF, 0.9958, better than the recommended R^2^ > 0.90.

Reproducibility and repeatability of the method of analysis was investigated by evaluating analytes quantitation (recovery) in five repeated measurements and over five days. The variances in concentrations in repeated measurement were used to define the method and instrument precision, which in all cases were <10%.

The recoveries of BaP and BkF were evaluated by spiking with internal standard. The spikes were recovered by extraction after homogenization and equilibration, and analysed. The recovery of BaP and BkF triplicate spikes were ranged 89.52–92.74% and 91.07–93.55%, with relative standard deviations (RSDs) ranging between 6.47–12.59% and 7.86–13.71%, respectively.

### 3.2. Results

Benzo[a]pyrene (BaP) and benzo[k]fluoranthene (BkF) was present at below detection limit to detectable concentrations in the polycyclic aromatic hydrocarbon extractions from the differently processed fish samples. The mean concentration of the fractions BaP and BkF detected in the extracts recovered from the fillets of the different fish samples were variable. The results of the separation and detection of BaP and BkF in the fried, grilled and boiled *Tyrsites atun* (snoek), *Seriola lalandi* (yellow-tail), *Merluccius poli* (hake) and *Bram a brama* (angel fish) fish samples are presented in Table 1.

The concentration of BaP in the different fried fish samples was <0.01–1.45 µg/kg (0.63 ± 0.57), <0.01–1.01 µg/kg (0.56 ± 0.45), <0.01–0.67 µg/kg (0.39 ± 0.26) and 0.26–1.12 µg/kg (0.74 ± 0.33) in *Tyrsites atun* (snoek), *Seriola lalandi* (yellow-tail), *Brama brama* (angel fish) and *Merluccius poli* (hake) respectively; while the concentration of BkF ranged <0.01–2.24 µg/kg (0.83 ± 0.83), <0.01–1.86 µg/kg (0.48 ± 0.38), <0.01–0.98 µg/kg (0.71 ± 0.36) and <0.01–0.89 µg/kg (0.46 ± 0.40), respectively.

Benzo[a]pyrene and BkF levels in grilled *Tyrsites atun* (snoek), *Seriola lalandi* (yellow-tail), *Brama brama* (angel fish) and *Merluccius poli* (hake) fish samples ranged <0.01–1.21 µg/kg (0.52 ± 0.47), <0.01–0.84 µg/kg (0.44 ± 0.36), <0.01–0.56 µg/kg (0.32 ± 0.22) and 0.22–0.99 µg/kg (0.62 ± 0.27); and <0.01–1.86 µg/kg (0.69 ± 0.69), <0.01–0.72 µg/kg (0.40 ± 0.32), <0.01–0.82 µg/kg (0.47 ± 0.37), 0.28–0.75 µg/kg (0.38 ± 0.33), respectively.

The concentration of the PAH fractions in the boiled fish extracts were ranged; BaP, <0.01–0.84 µg/kg (0.37 ± 0.33), <0.01–0.59 µg/kg (0.31 ± 0.25), <0.01–0.38 µg/kg (0.23 ± 0.15) and 0.31–0.65 µg/kg (0.43 ± 0.19) in *Tyrsites atun* (snoek), *Seriola lalandi* (yellow-tail), *Brama brama* (angel fish) and *Merluccius poli* (hake) respectively, and BkF, <0.01–1.30 µg/kg (0.49 ± 0.48), <0.01–0.50 µg/kg (0.34 ± 0.18), <0.01–0.58 µg/kg (0.33 ± 0.26) and <0.01–0.52 µg/kg (0.44 ± 0.09), respectively.

**Table 1:** Concentration (µg/kg) of BaP and BkF fraction in PAH extracts from the different fish samples processed by different method.

Sample Type	PAH Concentration in Fried Fish		PAH Concentration in Grilled Fish		PAH Concentration in Boiled Fish	
	BaP (µg/kg)	BkF (µg/kg)	BaP (µg/kg)	BkF (µg/kg)	BaP (µg/kg)	BkF (µg/kg)
Snoek (*Tyrsites atun*)						
Range	ND–1.45	ND–2.24	ND–1.21	ND–1.86	ND–0.84	ND–1.30
Mean ± Standard deviation	0.63 ± 0.57	0.83 ± 0.74	0.52 ± 0.47	0.69 ± 0.69	0.37 ± 0.33	0.49 ± 0.48
∑_2_PAH	1.46		1.21		0.86	
Yellow-tail (*Seriola lalandi*)						
Range	ND–1.01	ND–0.86	ND–0.84	ND–0.72	ND–0.59	ND–0.50
Mean ± Standard deviation	0.56 ± 0.45	0.48 ± 0.38	0.44 ± 0.36	0.40 ± 0.32	0.31 ± 0.25	0.34 ± 0.18
∑_2_PAH	1.04		0.92		0.65	
Angel fish (*Brama brama*)						
Range	ND–0.67	ND–0.98	ND–0.56	ND–0.82	ND–0.38	ND–0.58
Mean ± Standard deviation	0.39 ± 0.26	0.71 ± 0.36	0.32 ± 0.22	0.47 ± 0.37	0.23 ± 0.15	0.33 ± 0.26
∑_2_PAH	1.10		0.79		0.56	
Hake (*Merluccius poli*)						
Range	0.26–1.12	ND–0.89	0.22–0.94	0.28–0.75	0.31–0.65	ND–0.52
Mean ± Standard deviation	0.74 ± 0.33	0.46 ± 0.40	0.62 ± 0.27	0.38 ± 0.33	0.43 ± 0.19	0.44 ± 0.09
∑_2_PAH	1.20		1.10		0.87	

### 3.3. Concentration Levels of PAH in the Processed Fish

Nearly all food substances, especially fish, meat, cereal, edible seeds, vegetables, vegetable oil, *etc.* contain PAHs to a varying extent [21,22]. They are added to food by deposition from PAH polluted soil or air on crops and food substances and/or uptaken from polluted air. The concentration levels of PAHs in uncooked food are usually low and may range from 0.01–0.1 µg/kg [23]. Endogenous PAH levels in the raw fish ((N = 10) < detection limit (0.1 µg/kg)) are the residue from the metabolism of taken-up contaminated food, and or dermal exposure in aquatic habitats contaminated or polluted with varying concentration levels of PAH. Oost* et al.* [24] reported that the ability of fish and other vertebrates to metabolize PAHs results in their low concentration even in contaminated areas. Endogenous distribution of PAHs in fillets of the different fish samples may not be unconnected to the retention of metabolic spill over residues in the fat tissues of the different fish species. However, the elimination and remobilization of residues of PAHs depends on their solubility [25]. Raw fish in heavily polluted waters were reported to contain between 1.5 to 10.5 µg/kg of BaP [26].

Although a maximum concentration of BaP, 1.45 µg/kg, was observed in fillets of snoek (*Tyrsites atun*), fillet extracts of hake (*Merluccius poli*) had the highest mean BaP concentration (0.74 µg/kg). Also the maximum BkF concentrations of 2.24 µg/kg was measured in fillets of snoek (*Tyrsites atun*), and with a maximum mean levels of 0.83 ± 0.74 µg/kg. The sum of the two PAHs (∑_2_PAH)* i.e.*, BaP and BkF reached a maximum of 1.46 µg/kg in fried fish, with the least levels of 0.56 µg/kg in boiled angel fish (*Brama brama*). The order of abundance of the PAH fractions, BaP and BkF in the different fish were snoek (*Tyrsites atun*) > hakes (*Merluccius poli*) > yellow-tail (S*eriola lalandi*) > angel fish (*Brama brama*).

However, in home prepared fish and foods, and commercially available processed fish sold in eateries, the levels of BaP and BkF may be higher depending on the method used for processing, and the duration of thermal exposure. Reinik* et al.* [18] noted that the variations in PAHs levels in processed fish and meats were the result of several factors such as processing methods, fat content of the fish/food, temperature, processing time, distance from the heat source, fuel type, and oxygen availability. Since the within and between species fish fillets fats and lipids content are known to be variable, the levels and extent of saturation/unsaturation of fish fillets fats and lipids partly defines the variation in PAHs levels especially the endogenous amount formed via dehydrocyclization of long chain mono-unsaturated hydrocarbons during processing.

### 3.4. Effect of Processing Methods on PAHs Levels

The preparation and or processing of fish and fish products for edibility have been reported to result in the elevation of PAHs levels [13,27,28,29,30], largely by superficial sorption on fish skin and raised endogenous levels within fish fillets, as a result of the formation and deposition of PAH from thermal source, penetration of volatile, aromatization and or dehydrocyclization during heat treatments using methods such as smoking, frying, boiling, grilling, smoking, roasting* etc.* The concentrations of BaP and BkF detected in the fish fillets showed that, preparation/processing methods is related to their abundance. The sum of the two PAHs (∑_2_PAH)* i.e*.; BaP and BkF were 1.46, 1.21 and 0.86 µg/kg in the fried, grilled and boiled snoek (*Tyrsites atun*); 1.04, 0.92, and 0.65 µg/kg in the fried, grilled and boiled yellow-tail (*Seriola lalandi*); 1.10, 0.79 and 0.56 µg/kg in the fried, grilled and boiled Angel fish (*Brama brama*); and 1.20, 1.10, 0.87 µg/kg in the fried, grilled and boiled hake (*Merluccius poli*). The fried fish samples showed significantly higher (*p* < 0.05) levels of ∑_2_PAH, than grilled and boiled fish except fried and grilled yellow-tail (*Seriola lalandi*) and hake (*Merluccius poli*). The abundance of the PAH fractions in the fillets extracts of the processed fish samples is in the order of fried > grilled > boiled.

The higher than grilled and boiled PAHs levels in the fried fish samples may partly be attributed to the penetration and retention of vegetable oil used in frying, and this may lead to the elevation of PAHs beyond background concentration. The presence of PAHs in vegetable oils is as a result of its formation during the drying of seeds used in the production of crude vegetable oils [31,32]. The levels of PAH in edible vegetable oil depends upon the condition under which the refining and deodourisation procedures takes place, and the quality control measure put in place [33]. Repeated use of oil especially when in use for frying food substances such as fish, meat and other friable, may result in an increase in PAHs levels [34]. The levels of PAHs in grilled fish may depend on the thermal method employed in the grilling process [33]. Charcoal grills were reported to result in higher PAHs levels compared with oven grills. Oven grill and boiling can also results in variable PAHs levels depending of the temperature achieved, circulation of air and the grilling/boiling time [32].

The results from this study also revealed significant (*p* < 0.05) variable levels of BaP and BkF in the fillet extracts obtained from different fish species prepared by the same method except for grilled and boiled *Seriola lalandi* and boiled *Merluccius poli.* Generally, the concentration levels of BkF were slightly higher (*p* > 0.05) than BaP in all the fish species processed by boiling. However the levels of BkF in fried and grilled snoek (*Tyrsites atun*) and angel fish (*Brama brama*) fillets were significantly higher than BaP. The concentration of BaP in fillets of hake (*Merluccius poli*) on the other hand, was significantly (*p* < 0.05) higher than the levels of BkF. Also, BaP levels in fillets of yellow-tail (*Seriola lalandi*) are not significantly higher (*p* > 0.05) than BkF.

Consequently the method used for processing the fish samples to edibility may impact on them high concentration of PAHs, while the remobilization and distribution of PAH in the fish fillets may also be species variable

### 3.5. Dietary Safety Threshold and Toxicity

The concentration of BaP and BkF in the different fish processed by frying, grilling and boiling generally ranged <0.01–1.45 µg/kg and <0.01–2.24 µg/kg respectively. Hence consumers of fish and fish products processed using similar methods may be exposed to BaP and BkF within the measured range, although these may be higher. The levels of BaP reported in many studies varied between 0.05–60 µg/kg [35]. Levels of PAH fractions in meat, fish and other food substances have been reported severally; however data concerning PAH levels in fried fish, or other fried food substances are not as common. The results reported in earlier studies for BaP, and the total PAHs (∑_i_PAH) in some processed food types, prepared using different thermal methods are variable. The concentrations of BaP and BkF observed in the differently processed fish types in this study, were within the 0.03–100 µg/kg and 0.11–3.93 µg/kg, BaP reported by Simko and Janoszka* et al.* [36,37], respectively, for smoked and cooked meat. Many other study reports published higher BaP and ∑_i_PAH levels in different processed foods [38].

Concerns about the presence and accumulation PAHs in food substances arises from the reported observed health effects of its association with unsaturated and saturated fats [10,25]. The Food and Agricultural Organization and World Health Organisation Joint Expert Committee on Food Additives [39] listed food substances such as vegetable oil and fats, fish, meat and cereal products as major human dietary exposure routes to PAHs. Critical thresholds for food safety and health are not clearly defined due to the complex nature of PAHs, and because of the unlikely event of exposure to single PAH fractions. Therefore, the recommended guideline levels and critical thresholds for different PAH fractions in food matrices varies for different food substances, and also from one country to another. The critical threshold for fish and fish products therefore relies on the EU [40] and WHO recommendation of 5 and 10 µg/kg for BaP and ∑_i_PAH in smoked food substances. The sum of the two PAH fraction* i.e.*, BaP and BkF (∑_2_PAH) measured in fillets of each of the fried, grilled and boiled snoek (*Tyrsites atun*), angel fish (*Brama brama*), yellow-tail fish (*Seriola lalandi*) and hake (*Merluccius poli*) were lower than the 2 µg/kg recommended value suggested for BaP alone, in food substances processed by methods other than smoking [40].

The lipophilic character of PAHs and their tendency for bioaccumulation in blood lipids and fats, tissues and muscles, and in adipose and subcutaneous tissues in human body portend potential toxic consequence. Thus, there is the need to mitigate, or enforce reduced exposure to the barest minimum. Regional considerations may however arise, as a result of the variations in human response to different PAH fractions, and to residue levels. Therefore having uniform set of rule throughout the world may be misleading. The margin of exposure (MoE = BMDL_10_/dietary exposure) of each PAH fraction must therefore be determined with respect to metabolism. Such information will therefore provide an insight into dietary exposure risk involved in human consumption of fish and fishery products.

## 4. Conclusions and Recommendations

The different manipulation and thermal treatment* i.e.*, frying, grilling and cooking, used in the preparation and or processing of all the different fish samples for edibility, may result in the elevation of the PAH fractions BaP and BkF, although the fillet concentration levels were significantly variable over a considerable range between fish species, and between different processing methods. The fried fish showed higher levels of ∑_2_PAH, followed by grilled fishes and then boiled fish samples. However, the concentration levels of BaP and BkF detected in the fillets of the fried, grilled and boiled snoek (*Tyrsites atun*), angel fish (*Brama brama*), yellow-tail fish (*Seriola lalandi*) and hake (*Merluccius poli*) were lower than the recommended 5 µg/kg value for BaP in food substances, indicating that, there is no cause for concern, especially when the safety threshold of 2 µg/kg is enforced. The levels of BkF in most of the different processed fish were slightly higher than BaP levels, and this may be attributed to the different methods used in preparing the fish samples.

## References

[1] Lawrie R.A., Ledward D.A. (2006). Lawrie’s Meat Science.

[2] National Cattlemen’s Beef Association Don’t Miss out on the Benefits of Naturally Nutrient Rich Lean Meat. http://www.beef.org/uDocs/whatyoumisswithoutmeat638.pdf.

[3] Gehlhar M., Coyle W., Regmi A. (2001). Global food consumption and impacts on trade patterns. Changing Structure of Global Food Consumption and Trade.

[4] Hanley S.B. (1999). Everyday Things in Pre-Modern Japan: The Hidden Legacy of Material Culture.

[5] Karl H., Leinemann M. (2006). Determination of polycyclic aromatic hydrocarbons in smoked fishery products from different smoking kilns. Zeitschriftfur Lebensmittel Untersuchung und Forschung.

[6] Alcicek Z. (2011). Determination of shelf life and PAHs content of smoke anchovy (*Engrualis encrasicolus*, Linneeaus, 1758) nugget with different level liquid smoke flavours during chilled storage. J. Anim. Vet. Adv..

[7] Bartoszek A., Sikorski Z.E. (2007). Mutagenic, carcinogenic, and chemo-preventive compounds in foods. Chemical and Functional Properties of Food Components.

[8] Dunn B.P., Fee J. (2008). Polycyclic aromatic hydrocarbon carcinogens in commercial meat. J. Fish. Res. Board Can..

[9] IARC (2010). Some non-heterocyclic polycyclic aromatic hydrocarbons and some related exposures. IARC Monographs on the Evaluation of Carcinogenic Risks to Humans.

[10] Dennis M.J., Massey R.C., McWeeny D.J., Knowles M.E., Watson D. (1983). Analysis of polycyclic aromatic hydrocarbon in UK total diets. Food Chem. Toxicol..

[11] Dennis M.J., Massey R.C., Cripps G., Venn I., Howarth N., Lee G. (1991). Factors affecting the polycyclic aromatic hydrocarbon content of cereals, fats and other food products. Food Addit. Contam..

[12] Chen B.H., Wang C.Y., Chiu C.P. (1996). Evaluation of polycyclic aromatic hydrocarbons in meat products by liquid chromatography. J. Agric. Food Chem..

[13] Chen B.H., Lin Y.S. (1997). Formation of polycyclic aromatic hydrocarbons during processing of duck meat. J. Agric. Food Chem..

[14] Guillen M.D., Sopelana P., D’Mello J.P.F. (2003). Polycyclic aromatic hydrocarbons in diverse foods. Food Safety.

[15] Jira W. (2004). A GC/MS method for the determination of polycyclic aromatic hydrocarbon (PAH) in smoked meat products and liquid smokes. Eur. Food Res. Technol..

[16] European Commission (EC) (2005). Commission Recommendation of 4 February 2005 on the further investigation into the levels of polycyclic aromatic hydrocarbon in certain foods. Off. J. Eur. Union.

[17] European Commission (EC) (2005). Commission Regulation (EC) No 208//2005 of 4 February 2005 amending Regulation (EC) No 466/2001 as regards polycyclic aromatic hydrocarbons. Off. J. Eur. Union.

[18] Reinik M., Tamme T., Roasto M., Juhkam K., Tenno T., Kiis A. (2007). Polycyclic aromatic hydrocarbon (PAHs) in meat products and estimated intake by children and the general population in Estonia. Food Addit. Contam..

[19] Simko P., Gergely S., Karovica J., Drdak M., Knezo J. (1993). Influence of cooking on Benzo[a]pyrene content in smoked sausages. Meat Sci..

[20] Olatunji O.S., Fatoki O.S., Opeolu O.O., Ximba B.J. (2014). Determiantion of polycyclic aromatic hydroxcarbons (PAHs) in processed meat products using gas chromatography-flame ionization detector. Food Chem..

[21] Stolyhwo A., Sikorsi Z.E. (2005). Polycyclic aromatic hydrocarbons in smoked fish—A critical review. Food Chem..

[22] EFSA. Polycyclic Aromatic Hydrocarbons in Food, Scientific Opinion of the EFSA.

[23] Gomez-Guillen M.C., Gomez-Estaca J., Gimenez B., Montero P. (2009). Alternative fish species for cold-smoking process. Int. J. Food Sci. Technol..

[24] Oost Van der R., Beyer J., Vermeulen N.P.E. (2003). Fish bioaccumulation and biomarkers in environmental risk assessment: A review. Environ. Toxicol. Pharmacol..

[25] ATSDR (1999). Toxicological Profile: Screening Level Ecological Risk Assessment Protocol.

[26] Anyakora C., Arbabi M., Coker H. (2008). A screen for benzo(a)pyrene in fish samples from crude oil polluted environments. Am. J. Environ. Sci..

[27] Mottier P., Parisod V., Turesky R.J. (2000). Quantitative determination of polycyclic aromatic hydrocarbons in barbecued meat sausages by gas chromatography coupled to mass spectrometry. J. Agric. Food Chem..

[28] Rey-Salgueiro L., Garcia-Falcon M.S., Soto-Gonzalez B., Simal-Gandara J. (2009). Occurrence of polycyclic aromatic hydrocarbons and their hydroxlated metabolites in infant foods. Food Chem..

[29] Garcia-Falcon M.S., Simal-Gandara I. (2005). Polycyclic aromatic hydrocarbons in smoked from different woods and their transfer during traditional smoking into chorizo sausages with collagen and tripe casings. Food Addit. Contam..

[30] Gomez-Estaca J., Gómez-Guillen M.C., Montero P., Sopelana P., Guillen M.D. (2011). Oxidative stability, volatile components and polycyclic aromatic hydrocarbons of cold-smoked sardine (*Sardina pilchardus*) and dolphin fish (*Coryphaena hippurus*). Food Sci. Technol..

[31] El Badry N. (2010). Effect of household cooking methods and some food additives on polycyclic aromatic hydrocarbons (PAHs) formation in chicken meat. World Appl. Sci. J..

[32] Lacoste F. (2014). Undesirable substances in vegetable oils: Anything to declare?. Qual. Food Saf..

[33] FAO (1994). Processing and refining edible oil: Fat and oil in human nutrition. Report of a Joint Expert Consultation of Food and Agriculture Organization Corporate Document Repository.

[34] WHO (2006). Polycyclic Aromatic Hydrocarbons. WHO Food Additive Series 55: Safety Evaluation of Certain Contaminants in Food.

[35] Lawrence J.F., Weber D.F. (1984). Determination of polycyclic aromatic hydrocarbons in some Canadian commercial fish, shellfish, and meat products by liquid chromatography with confirmation by capillary gas chromatography-mass spectrometry. J. Agric. Food Chem..

[36] Simko P. (2002). Determination of polycyclic aromatic hydrocarbons in smoked meat products and smoked flavouring food additives. J. Chromatogr. B.

[37] Janoszka B., Warzecha L., Blaszczyk U., Bodzek D. (2004). Organic compounds formed in thermally treated high protein food. Part 1: Polycyclic aromatic hydrocarbons. Acta Chromatogr..

[38] Phillips D.H. (1999). Polycyclic aromatic hydrocarbons in the diet. Mutat. Res..

[39] JECFA Joint (FAO/WHO JECFA) Expert Committee on Food Additives Sixty-Fourth Meeting.

[40] European Commission (EC) (2006). Commission Regulation (EC) No. 1881/2006 setting maximum levels for certain contaminants in food stuffs. Off. J. Eur. Union.

